# Research on Price Influencing Factors of Third-Party Payment Platforms: An Empirical Study From China

**DOI:** 10.3389/fpsyg.2022.829568

**Published:** 2022-08-02

**Authors:** Na Wang, Wen Liu, Jinping Shi

**Affiliations:** School of Business, Hubei University, Wuhan, China

**Keywords:** bilateral market, third-party payment platform, pricing factors, empirical study, Chinese payment platforms

## Abstract

In this article, we use the two-sided market theory as a support and take the operating data of eight Chinese third-party payment platform companies as samples, based on pricing models and multiple regression analysis, to build a price feature model for a third-party payment platform. The results show that under the two-sided market environment, the scale of consumer and the same-side network externality of the merchant have a negative significant effect on the pricing of the third-party payment platform to the seller; The market share of the platform and the bank fee cost of the platform have a positive significant effect on the pricing of a third-party payment platform to the merchant. At the same time, the same-side network externality of the seller, the scale of a merchant, and the selection of the business model of the platform have no significant effect on the pricing of the third-party payment platform to the merchant. These conclusions provide a scientific basis for a third-party payment platform enterprise to develop an appropriate pricing strategy and operating model.

## Introduction

People with different beliefs and backgrounds are interacting due to the world’s rapid globalization (Kharroubi, 2021). Ionescu (2021) empirically examines corporate environmental performance, climate change mitigation, and green innovation behavior in sustainable finance. Similarly, Konhäusner et al. (2021) pointed out that the production and release of independent print media poses significant hurdles in terms of financing. And Kovacova et al. (2020) and Kovacova and Lewis (2021) draw on a substantial body of theoretical and empirical research on smart factory performance, cognitive automation, and industrial big data analytics in sustainable manufacturing Internet of Things. Digitalization which have been intensified by the COVID-19 pandemic and present great opportunities for economic growth and the development of businesses (Malkawi and Khayrullina, 2021).

At the same time, it brings challenges such as new requirements for human skills (Malkawi and Khayrullina, 2021). And the COVID-19 pandemic has also reshaped customer attitudes, behaviors, values, and expectations, reconfiguring consumer traits, sentiments, trust, and engagement, thus leading to altered purchasing decisions and habits, and buying patterns in terms of psychological risk perception (Watson and Popescu, 2021). Rydell and Kucera (2021) also explored cognitive attitudes, behavioral choices, and purchasing habits during the COVID-19 pandemic. Just as Drugãu-Constantin (2019) and Miricã (2019) synthesized existing studies and investigated the neural correlates of consumer behavior. After more than 15 years of rapid development, third party payment institutions have become an important supplement to the service market in China (Pu et al., 2018).

With the development of internet technology, e-commerce and the rise of the “house economy,” online shopping has become the preferred consumption mode for consumers. Barbu et al. (2021) declared that perceived value, customer support, assurance, speed and perceived firm innovativeness are positively related to customer experience in fintech. With the rapid advent of e-commerce in China, the technological innovation of third-party payment has experienced explosive growth. This important technological innovation, initiated by emerging Internet companies, is helping the traditional financial industry’s payment business—represented by commercial banks—expand in both depth and breadth (Yao et al., 2018). Most of the payment models supported by emerging technologies are centered on platform enterprises. Platform enterprises take the bilateral market as the micro-basis for their existence, and attract bilateral market participants to the platform by setting transaction rules (price structure) and competitive strategies. Under the two-sided market environment, network externality generally exists. This externality depends not only on the number of participants on the same side of the market, but also on the number of participants on the other side of the market, that is, “indirect network externalities.” This strong network externality between the two sides of the market has completely subverted the traditional enterprise value creation model, so third-party payment platform companies need to pay attention to both sides of the access market. In the context of rapid changes in information technology, how to correctly build the business of third-party payment platform enterprises, so as to seek a more appropriate business model and seize the opportunity in the fierce international competition, has become a key problem that urgently needs to be solved by the theoretical and practical circles. Hence, we study the pricing factors for third-party payment empirically, which lay a solid theoretical foundation for the third-party payment platform enterprise to formulate a suitable price strategy.

## Literature Background

With the development of the two-sided market theory, the “black box” of third-party payment platform enterprises has been gradually opened. Most scholars believe that a “platform” refers to a third-party access system that facilitates bilateral (or multilateral) transactions and obtains revenue from them (Eisenmann et al., 2006). If the platform can change the transaction volume by increasing the charges to one side while reducing the charges to the other side to the same extent, this platform market is called a “two-sided market” (Charles and Tirole, 2006). Under the two-sided market environment, the research on the pricing strategy of the third-party payment platform has become another focus of current research. Erliang (2010) found that the transaction fees charged by the platform to bilateral users are highly correlated with network externalities, and the network externalities between groups are important factors restricting the predatory pricing of third-party payment monopoly platforms. At the same time, the platform’s pricing and profit are positively related to the probability of successfully matching users. Subsequently, Ling (2012) found that the platform is likely to set the price below the marginal cost for consumers, even zero price or negative price. But the pricing of merchants on the platform is inversely related to the transaction amount, that is, the platform charges lower fees to merchants with a larger transaction amount, while charging higher fees to merchants with a lower transaction amount. As the number of transactions by users or the platform improves the technology of successful transactions by both parties, the platform will choose to adopt the pricing strategy of low registration fees and a high transaction fees for merchants. Ling and Shuai (2014) pointed out that the increase in any of these variables, including the self-network externality of users on either side, the cross-network externality between users on both sides, the number of transactions occurred, and the user’s preference for the platform, will cause the platform to reduce the handling fees for consumers and registration fees for merchants, thereby increasing the handling fees for merchants to transform two-step charging system established early by the platform for merchants gradually into a way of only charging handling fees for merchants. Lili (2016) pointed out that transfer cost is an important factor that affects the initial pricing, secondary pricing, and profit of the platform. At the same time, platform competitors can gain a dominant position only if they form an effective transfer cost constraint on users. During the same period, Fumao et al. (2016) show that third-party payment platforms need to follow the first mover (bank) as a follower when setting user prices, not only to consider the demand elasticity of consumers and merchants and the demand elasticity of the entire platform, but also to consider the expenditure on bank side (that is, the fee charged by the bank to the platform), and it is basically positively related to the platform pricing. In the near future, Burcu et al. (2020) suggested that considering integration investment can create market regimes in which the standard pricing results from the extant platform literature no longer hold. Therefore, integration investments must be well-coordinated with pricing decisions made for both sides of the market. These works mostly analyzed the factors influencing the price of third-party payment platforms from the theoretical perspective, but failed to analyze the main factors that should be considered in the pricing of third-party payment platforms from the practical perspective.

In view of this, we take the two-sided market theory as the support, and the operating data of eight third-party payment platform companies from China as samples, based on the pricing model and multiple regression model to study the main factors that need to be considered when determining the price of the third-party payment platform. And further, we discuss the decisive factors that have a significant impact on the pricing of the third-party payment platform. Based on this, the pricing factor model of the third-party payment platform is finally constructed.

## Data and Methodology

In the actual operation of the third-party payment platform, it has always been free for consumers, and at the same time adopted a gradual transition from a free to fee-based pricing strategy for merchants. Therefore, this article mainly studies the factors that influence the pricing of third-party payment platforms for merchants.

### Research Hypothesis

#### The Relationship Between the Scale of Users and Platform Pricing to Merchant

Hanlin (2006) proposed that for the monopoly of bilateral platforms, no matter the mode of the transaction fee, registration fee, or two-step fee, the number of users has a reverse relationship with the price set by the platform, and when the number of users is large, the platform will also charge zero price or negative price to users. Similarly, Chenchen (2013) pointed out that regardless of the pricing method adopted by the platform, the size of the user is an important factor affecting the platform pricing and basically has a reverse change relationship with the platform’s pricing.

Based on the above-mentioned analysis, we make the following assumptions:

H_1:_ The consumer scale of the third-party payment platform is negatively correlated with the platform’s pricing to the merchant, and the former has a significant influence on the latter.

H_2:_ The merchant scale of the third-party payment platform is negatively correlated with the platform’s pricing to the merchant, and the former has a significant influence on the latter.

#### The Relationship Between the Same-Side Network Externality of Users and the Platform Pricing to Merchant

Guisun (2010) pointed out that in the case of multi-platform access of merchants, intra-group network externality was negatively correlated with platform pricing. Le (2017) showed that the platform’s pricing to bilateral users is not only inversely proportional to the strength of inter-group network externality of users, but also negatively correlated with the strength of intra-group network externality of users.

Based on the above-mentioned analysis, we make the following assumptions:

H_3_: There is a negative correlation between the same-side network externality of consumers on the third-party payment platform and the platform’s pricing to the merchant, and the former has a significant influence on the latter.

H_4:_ There is a negative correlation between the same-side network externality of merchants on the third-party payment platform and the platform’s pricing to the merchant, and the former has a significant influence on the latter.

#### The Relationship Between the Market Share of Platform and the Platform Pricing to Merchant

Through a balanced analysis of the two-stage bilateral platform Hotelling competition model, Na (2008) pointed out that platforms with a higher (lower) bilateral market share in the first period will usually adopt higher (lower) prices to users in the second period. Similarly, Ling (2012) reached a similar conclusion. She pointed out that when users choose to register platform for the second time, the platform that occupies a larger market share (i.e., has accumulated more registered users) in the early stage can improve the pricing to users, that is, the market share of the platform is positively correlated with the pricing of the platform to users.

Based on the above-mentioned analysis, we make the following assumption:

H_5:_ There is a positive correlation between the market share of third-party payment platforms and the platform’s pricing to the merchant, and the former has a significant influence on the latter.

#### The Relationship Between the Bank Fee Cost of the Platform and the Platform Pricing to Merchant

Shasha (2011) pointed out that in the case of a multi-period game between the bank and the third-party payment platform, for the cost of the previous bank fee, the platform will generally increase the transaction fee charged to users and formulate an appropriate allocation structure according to the size of demand price elasticity of users on both sides, so as to transfer the bank cost to consumers and merchants, respectively. Similarly, Fumao et al. (2016) pointed out that the fees charged by the bank to the platform were basically positively correlated with the platform’s pricing to users.

Based on the above-mentioned analysis, we make the following assumption:

H_6:_ There is a positive correlation between the bank fee cost of the third-party payment platform and the platform’s pricing to the merchant, and the former has a significant impact on the latter.

#### The Relationship Between the Selection of Business Model of the Platform and the Platform Pricing to Merchant

Hanlin and Guan (2008) studied the pricing strategies of the two-sided market platforms under the vertically integrated structure, and found that whether it is a monopoly platform or a competitive platform, vertically integrated platforms generally reduce the pricing to users. Linlin (2013) believes that the vertically integrated model and vertically separated model of the third-party payment platform will have different impacts on the differentiation degree of products or services provided by the platform, user attribution, and platform pricing model, which will ultimately have different impacts on the pricing of users.

Based on the above-mentioned analysis, we make the following assumption:

H_7:_ The selection of the business model for a third-party payment platform is related to the platform’s pricing for the merchant, and the former has a significant influence on the latter.

### Date

We selected eight domestic mainstream platforms with the largest market share in third-party mobile payment in China, including Alipay, WeChat Pay, Lakala, Quick Money, BESTPAY, One Wallet, Baidu Wallet, and JD Pay. We use quarterly panel data from the third quarter of 2014 to the fourth quarter of 2017 as the source of the original data (the total market share of these eight third-party payment platforms is between 90 and 95%, indicating that the market size is large enough). The data have a total of eight groups, and each group has a total of 112 observations.

According to the relevant data from iResearch, the domestic third-party mobile payment market showed a trend toward ultra-fast growth in 2014, and the overall transaction size was 599.247 billion yuan in China, an increase of 391.3% compared with the previous year. At the same time, a large number of domestic consumers shifted from the PC to the mobile terminal, and many third-party mobile payment platforms also appeared, such as WeChat Pay, One Wallet, Baidu Wallet, and so on. Therefore, we choose the third quarter of 2014 as the starting point of the research period to make the sample data. The relevant data are mainly derived from the financial statements of the listed companies affiliated with each platform and the statistical data of the third-party payment industry released by Internet consulting agencies (including Analysys, iResearch, and BigData Research). Some unavailable data are processed with missing values using appropriate interpolation methods in advance.

We use panel data for empirical analysis, which generally improves the freedom of the regression model. It can solve the problem of missing variables caused by unobservable individual differences or “heterogeneity” and increase the total sample size, reducing the effect of multicollinearity among explanatory variables, so as to obtain a more accurate parametric estimated value.

### Variables

#### The Dependent Variable

Platform pricing for merchants (y_*it*_) stands for the pricing of the platform to merchants. Most domestic third-party payment platforms mainly charge merchants a certain percentage of transaction fee (or transaction commission), which is usually equal to the transaction value multiplied by the transaction rate of the payment products (or the instant account products) stipulated by the platform. Some platforms also charge a certain access fee, that is, the fee that merchants need to pay for the first access to the platform system, which is generally a one-time fee. The price ranges from several hundred yuan to several thousand yuan, and the service period is usually 1 year. However, as the competition in the third-party payment market continues to intensify, there are only a few platforms that charge this fee, and most of them focus on the subsequent transaction fees. Currently, third-party payment platforms mainly provide five kinds of payment products, which are computer website payment, mobile web payment, mobile app payment, scan code payment, and barcode payment. The detailed description is presented in Table 1.

**TABLE 1:** Instructions for the main payment products of the third-party payment platform in China.

Payment products	Application (use) scene description
Computer website payment	The buyer pays on the merchant’s computer website and directly selects the corresponding third-party payment module to complete the payment.
Mobile web payment	The buyer pays on the merchant’s mobile website, evokes a third-party payment application through the browser to make the payment and can continue to use the webpage to complete the payment without the third-party payment application.
Mobile app payment	The buyer pays in the merchant’s mobile app, directly selects the corresponding third-party payment module (the merchant integrates and open SDK in the app and activates it) to complete the payment, or can directly purchase goods and services in the third-party payment app and complete the payment.
Scan code payment	Offline buyers complete payment by using third-party payment applications or related applications or scanning the merchant’s QR code.
Barcode payment	The merchant directly scans the (pay code) barcode or QR code in the third-party payment application of the offline buyer to directly insert the buyer’s transaction funds into the seller’s account.

*Source: Organized by the author.*

Many platforms offer different rates for different industry categories, such as Alipay stipulates that the fee rate for general industries is 0.6%, the fee rate for special industries (such as digital entertainment, games, 3C digital, etc.) is 1.0%, the fee rate for physical business categories stipulated by WeChat Pay is 0.6%, and the fee rate for the virtual business category is 1.0% in China. Therefore, we choose the average fee rate of payment products on the third-party payment platform to represent the pricing of the platform to the merchant. Among them, the fee rate of payment products mainly comes from the merchant charge rules or standards published on the official website of each third-party payment platform and the corresponding merchant charge adjustment announcement.

#### The Independent Variables

The consumer scale (x_1*it*_) represents the scale of consumers, and the unit is 10,000 people. It mainly uses the total number of active consumers on the mobile application of each third-party payment platform to represent its value. Its data are mainly derived from the quarterly total number of active users of each third-party payment platform monitored and counted by Aanalysys’ big data application library. For third-party payment platforms, the number of active users mainly refers to those users who often use the platform to make payments and realize transaction activities, and their scale can play a certain network effect. The third-party payment platform should refer to and consider the number of active consumers rather than the total number of registered users when pricing to the merchant.

Consumer’s same-side (intra-group) network externality (x_2*it*_) represents the same-side (intra-group) network externality of consumers. Its size can directly affect the number of consumers on the same side. According to the definition of the strength of the same-sided network externality, x_2*it*_ = △x_2*it*_/x_2it–1_. Among them, △x_2*it*_ represents the amount of change in the consumer scale, which is calculated by subtracting the data of the previous year from the following year and then dividing the data of the previous year to finally obtain the variable data.

The merchant scale (x_3*it*_) represents the scale of the merchant, and the unit is 10,000 people. The value is mainly expressed by the total number of merchants connected to each third-party payment platform. Part of the data comes from relevant operational data published in the quarterly and annual financial statements of the listed companies to which each third-party payment platform belongs. The other part of the data comes from the relevant statistical data in the third-party payment industry research report released by the e-commerce research center and BigData consulting agency. There are still some missing values in the data, so the data was processed before the empirical analysis, and the mean value was mainly used for interpolation.

A merchant’s same-sided network externality (x_4*it*_) represents the same-sided network externality of the merchant, and its strength can directly affect the total number of merchants on the same side. According to the definition of network externality strength, x_4*it*_ = △x_4*it*_/x_4*it*–1_. Among them, △x_4*it*_ represents the amount of change in the scale of the merchant, which is calculated by subtracting the data of the previous year from the following year and then dividing the data of the previous year to finally obtain the variable data.

Market share of platform x_5*it*_ represents the market share of third-party payment platforms. It mainly refers to the proportion of the mobile payment (consumption) scale of each platform to the total transaction size of the entire third-party mobile payment market. The data come mainly from the quarterly and annual research reports on the third-party payment market between 2014 and 2017 released by iResearch.

The bank fee cost of the platform (x_6*it*_) represents the cost of the bank fee for the third-party payment platform, and the unit is 100 million yuan. And x_6*it*_ = T_it_*R_*it*_, where T_*it*_ represents the mobile consumption scale of each platform. The data are mainly derived from the specific transaction scale values published in the quarterly and annual financial statements of the listed companies. The data are also obtained by multiplying the overall transaction size of the third-party payment industry and the proportion of platforms in each quarter released by Analysys. Besides, R_*it*_ indicates the platform’s charges rate for the transfer of funds stipulated by the bank. Since most third-party payment platforms are connected to more than one bank and each bank also charges different rates, we use the general transfer rate of 0.2% stipulated by banks on the market for most third-party payment platforms as the standard to calculate the platform’s bank charges cost.

The selection of the business model of the platform (x_7*it*_) represents the selection of the business model of the third-party payment platform and is represented by dummy variables. If the platform adopts an integrated business model, it will be represented by “1”; If the platform adopts a vertically separated business model, it will be represented by “0.” For example, Alipay has been connected to the Taobao platform since its establishment, while Lakala directly cooperates with various merchants to provide them with professional acquiring or payment services.

### Model Description

Based on the general pricing theory, we take the pricing of third-party payment platform enterprises to access merchants as a function of influencing factors, and construct a multiple linear regression model to verify the hypothesis proposed above. The specific model is as follows:

$$y_{i\,t}=\alpha +\beta_{1}\,x\,1_{i\,t}+\beta_{2}\,x\,2_{i\,t}+\beta_{3}\,x\,3_{i\,t}+\beta_{4}\,x\,4_{i\,t}+\beta_{5}\,x\,5_{i\,t}+\beta_{6}\,x\,6_{i\,t}+\beta_{7}\,x\,7_{i\,t}+\varepsilon_{i\,t}$$

(1) (i=1,2,…,8;t=1,2,…,14)

**FIGURE 1:**
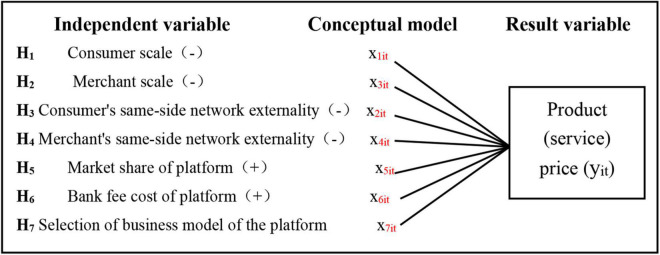
Research framework.

Among them, y_*it*_ represents the unit price of the product (service) *i* provided by the platform to the merchant (seller) at time *t*. It is a function of the consumer scale, the same-side network externality of consumers, the merchant scale, the same-side network externality of merchants, the market share of the third-party payment platform, the bank charges cost of the third-party payment platform, and the selection of the business model by the third-party payment platform. x_1*it*_ represents the number of consumers (buyers) who access the platform at time *t* and use the product (service) *i* provided by the platform. x_2*it*_ represents the strength of the network externality generated by consumers (buyers) accessing the platform at time t and using product (service) *i* provided by the platform to other consumers (buyers) using the platform. x_3*it*_ represents the number of merchants (sellers) who access the platform at a time t and use the product (service) *i* provided by the platform. x_4*it*_ represents the strength of the network externality generated by merchants (sellers) accessing the platform at a time t and using the product (service) *i* provided by the platform to other merchants (sellers) using the platform. x_5*it*_ represents the market share of product (service) *i* provided by the platform at time *t*. x_6*it*_ represents the bank charges cost of the product (service) *i* provided by the platform at time *t*. x_7*it*_ represents the business model of the platform providing products (services) *i* at time t. If the platform adopts an integrated business model, it will be represented by 1. If a vertically separated business model is selected, it will be represented by 0. α and β represent the intercept term and regression coefficient, respectively. ε_*it*_ represents the random error term, i represents various platforms, and t represents the period. The research framework is shown in Figure 1.

## Analysis of Results

### Data Description and Statistical Analysis

Since the variable values of the consumer scale (x_1*it*_) and the merchant scale (x_3*it*_) are large and the gap between the observations of other variables is also large, we first perform natural logarithm processing on these two variables. This can not only narrow their value range, but also ensure that the coefficients of the estimated equation are not too small or too large, making it easy to write and explain their economic meaning. In addition, natural logarithm processing can also eliminate the effects of heteroscedasticity and dimension without changing its economic nature. For example, 4,000/20 = 200, the result is larger, but ln4000/ln20 = 2.7686, where the data are more stable and the collinearity of the model is weakened. Therefore, we first perform natural logarithm processing on these variables and then perform a descriptive analysis of the data. The statistical results are presented in Table 2.

**TABLE 2:** Descriptive statistical results of data.

	y_*it*_	x_1*it*_	x_2*it*_	x_3*it*_	x_4*it*_	x_5*it*_	x_6*it*_	x_7*it*_
Mean	1.141582	7.223461	0.142893	4.973200	0.177194	11.63509	3.773302	0.651786
Median	0.810000	6.463594	0.086235	5.441908	0.115614	0.795000	0.271660	1.000000
Maximum	3.450000	11.49963	1.526686	7.161638	1.329501	82.60000	55.50800	1.000000
Minimum	0.000000	4.152142	–0.351870	1.543298	–0.121160	0.200000	0.011020	0.000000
Std. Dev.	0.952787	2.183924	0.266275	1.443380	0.196829	21.63884	8.861637	0.478545
Skewness	1.629709	0.653572	2.225928	–0.621153	2.975913	1.896369	3.385949	–0.637214
Kurtosis	4.273086	2.030500	10.61422	2.319696	15.57507	5.310866	15.89987	1.406041
Jarque-Bera	57.14122	12.35992	363.0451	9.361980	903.2642	92.04979	990.5717	19.43606
Probability	0.000000	0.002071	0.000000	0.009270	0.000000	0.000000	0.000000	0.000060
Sum	127.8571	809.0276	16.00399	556.9984	19.84572	1303.130	422.6098	73.00000
Sum Sq. Dev.	100.7662	529.4171	7.870155	231.2514	4.300321	51974.55	8716.675	25.41964
Observations	112	112	112	112	112	112	112	112

*Source: The author compiled the analysis results based on Eviews9.0.*

From Table 2, we can observe that the market share of the platform (x_5*it*_) is 82.6% at the maximum and 0.2% at the minimum, which indicates that the market share of each third-party payment platform is quite different. In addition, there is a large gap between the bank fee costs of various platforms. Also, we can observe that the standard deviation of the variables of consumer scale (x_1*it*_), merchant scale (x_3*it*_), market share of the platform (x_5*it*_), and bank fee costs of the platform (x_6*it*_) are all greater than 1, indicating there is a big difference between them. The standard deviation of variables such as platform’s pricing to merchants (y_*it*_), same-side network externality of consumers (x_2*it*_), same-side network externality of merchants (x_4*it*_), and selection of the business model of the platform (x_7*it*_) is less than 1. It shows that the difference between them and the average is small, so there are no abnormal fluctuation data and the data are relatively stable.

### Stationary Test of Variables

We use Eviews9.0 to perform unit root tests on panel data. Next, the ADF-Fisher method is used to test the unit root of the original sequence and the first-order difference sequence of each variable (with the dummy variable removed), and determine whether each variable is a stationary sequence based on the ADF statistics and the specific size of the corresponding P-value. The test results are presented in Table 3.

**TABLE 3:** ADF statistics and P-value results of unit root test of panel data.

	Level			1st diff.			Result
	I	I&T	N	I	I&T	N	
x1_*it*_	38.7002 (0.0012)	10.1386 (0.8593)	3.71699 (0.9993)	29.8352 (0.0189)	25.8577 (0.0561)	61.2465 (0.0000)	Stable
x2_*it*_	59.5456 (0.0000)	45.8197 (0.0001)	91.9762 (0.0000)	80.4984 (0.0000)	72.5437 (0.0000)	101.388 (0.0000)	Stable
x3_*it*_	81.6995 (0.0000)	43.3883 (0.0002)	3.02074 (0.9998)	63.5300 (0.0000)	68.8705 (0.0000)	65.2996 (0.0000)	Stable
x4_*it*_	62.8210 (0.0000)	56.2743 (0.0000)	85.8125 (0.0000)	108.876 (0.0000)	89.6275 (0.0000)	117.202 (0.0000)	Stable
x5_*it*_	22.0396 (0.1419)	24.2582 (0.0840)	42.6761 (0.0003)	66.7001 (0.0000)	62.6563 (0.0000)	77.9198 (0.0000)	Stable
x6_*it*_	0.67134 (1.0000)	7.50180 (0.9623)	0.34514 (1.0000)	52.8190 (0.0000)	59.3464 (0.0000)	66.7970 (0.0000)	Integrated of order
y_*it*_	3.53725 (0.9658)	17.7007 (0.2208)	22.0349 (0.0149)	42.4358 (0.0001)	32.8163 (0.0031)	46.8771 (0.0000)	Stable

*Source: The author compiled the analysis results based on Eviews9.0.*

We can observe that the bank fee cost of the platform (x_6*it*_) is a first-order integration sequence, while other variables are stationary sequences (or zero-order integration sequences), and all variables show non-same-order integration. Neither the co-integration test nor the regression of the original variable sequence can be performed directly, but the difference or logarithm of x_6*it*_ should be differentiated into the same order sequence while keeping the economic significance of the variable unchanged. If all variables are stationary sequences, the transformed sequence can be directly used for regression. Through the natural logarithm processing of x_6*it*_ and then the unit root test, the test results of the intercept term and trend term under the horizontal sequence are obtained. The statistical value of ADF is 65.4246, and the corresponding P-value is 0.0000. Therefore, x_6*it*_ is a stationary sequence. So the variables of the panel data are stationary series, and then the regression analysis of the panel data can be performed.

### Regression Analysis

We will perform F-test and Hausman test to select and determine the final model of panel data.

#### F-Test

We first choose a mixed estimation model or a fixed-effect model. Generally, F-test is used to determine it. The original hypothesis of the test is that the mixed estimation model is valid, that is, the mixed estimation effect model is accepted, and the alternative hypothesis of the fixed-effect model is valid. We first perform a mixed estimation model regression on the panel data. The detailed regression results are presented in Table 4.

**TABLE 4:** Regression results of mixed estimation model.

Variable	Coefficient	Std. error	t-statistic	Prob.
C	3.437036	0.583465	5.890737	0.0000
X1?	–0.278731	0.066042	–4.220536	0.0001
X2?	0.697321	0.348570	2.000518	0.0481
X3?	–0.070460	0.077172	–0.913033	0.3633
X4?	–0.512173	0.508597	–1.007032	0.3163
X5?	0.015974	0.006768	2.360332	0.0201
X6?	0.138788	0.082918	1.673798	0.0972
X7?	–0.012344	0.209937	–0.058800	0.9532
R-squared	0.232311	Mean dependent var		1.141582
Adjusted R-squared	0.180639	S.D. dependent var		0.952787
S.E. of regression	0.862449	Akaike info criterion		2.610668
Sum squared resid	77.35715	Schwarz criterion		2.804847
Log likelihood	–138.1974	Hannan-Quinn criter.		2.689453
F-statistic	4.495924	Durbin-Watson stat		0.085358
Prob(F-statistic)	0.000208			

*Source: The author compiled the analysis results based on Eviews9.0.*

Among them, the sum of squared residual (Sum squared resid) of the mixed estimation model is 77.35715, which is denoted as SSEr. The individual fixed-effect model regression is performed on the panel data. The detailed regression results are presented in Table 5.

**TABLE 5:** Regression results of individual fixed effect model.

Variable	Coefficient	Std. error	t-statistic	Prob.
C	2.234057	0.362995	6.154511	0.0000
X1?	–0.235485	0.054401	–4.328700	0.0000
X2?	–0.031387	0.084812	–0.370074	0.7121
X3?	0.029118	0.056997	0.510879	0.6106
X4?	–0.247604	0.122483	–2.021538	0.0460
X5?	0.029377	0.003690	7.960365	0.0000
X6?	0.068314	0.032365	–2.110754	0.0374
X7?	0.171887	0.103040	1.668156	0.0985
**Fixed Effects (Cross)**				
1–C	–0.447543			
2–C	0.461150			
3–C	–0.517797			
4–C	–0.432042			
5–C	–0.406028			
6–C	2.102745			
7–C	–0.136608			
8–C	–0.623878			

**Effects specification**				

**Cross-section fixed (dummy variables)**				

R-squared	0.964354	Mean dependent var		1.141582
Adjusted R-squared	0.959210	S.D. dependent var		0.952787
S.E. of regression	0.192431	Akaike info criterion		–0.334091
Sum squared resid	3.591872	Schwarz criterion		0.029994
Log likelihood	33.70910	Hannan-Quinn criter.		–0.186370
F-statistic	187.4453	Durbin-Watson stat		1.528659
Prob (F-statistic)	0.000000			

*Source: The author compiled the analysis results based on Eviews9.0.*

### Individual Fixed-Effect Model

Similarly, the sum of squared residual (Sum squared resid) of the individual fixed-effect model is 3.591872, which is denoted as SSEu. Then calculate the F-statistic according to the formula F = [(SSEr – SSEu)/(N-1)]/[SSEu/(NT-N-K)], where T is the number of time periods, K is the number of explanatory variables, and N is the number of individuals. Finally, the F-statistic is 284.58, which is greater than F_0.05_ (N-1, NT-N-K) = F_0.05_ (7, 97). So, the null hypothesis should be rejected, and the panel data should establish an individual fixed-effect model.

#### Hausman Test

Next, the panel data fixed-effect model and random effect model are selected. We mainly use the Hausman test to judge. According to the test results presented in Table 6, the chi-square statistic is 1,992.062077, and the corresponding P-value is 0.0000. Therefore, the null hypothesis is rejected at the 1% significance level, that is, the panel data in this article are suitable for fixed-effect model estimation.

**TABLE 6:** Hausman test results.

Correlated Random Effects – Hausman Test			
**Pool: POOL1**			

**Test cross-section random effects**			

**Test summary**	**Chi-Sq. statistic**	**Chi-Sq. d.f.**	**Prob.**

Cross-section random	1992.062072	7	0.0000

*Source: The author compiled the analysis results based on Eviews9.0.*

#### Regression Results

When the F-test and Hausman test are performed separately, it is finally determined that the panel data should be estimated using the individual fixed-effect model, since the individual fixed-effect model provides different intercept terms for different individuals. The intercept items corresponding to the eight third-party payment platforms and the final regression results of the panel data are detailed in Tables 7, 8, respectively.

**TABLE 7:** Intercept items corresponding to the eight third-party payment platforms.

*i*	*c*	Cross (FEM)	*c*_*i*_ = c + cross
1	2.234057	–0.447543	1.786514
2	2.234057	0.461150	2.695207
3	2.234057	–0.517797	1.71626
4	2.234057	–0.432042	1.802015
5	2.234057	–0.406028	1.828029
6	2.234057	2.102745	4.336802
7	2.234057	–0.136608	2.097449
8	2.234057	–0.623878	1.610179

*Source: The author compiled the analysis results based on Eviews9.0.*

**TABLE 8:** Regression results of panel data.

Variable	c_*i*_		Coefficient	Std. error	t-statistic		Prob.
x1_*it*_	1.786514		–0.235485	0.054401	-4.328700		0.0000
x2_*it*_	2.695207		–0.031387	0.084812	-0.370074		0.7121
x3_*it*_	1.71626		0.029118	0.056997	0.510879		0.6106
x4_*it*_	1.802015		–0.247604	0.122483	-2.021538		0.0460
x5_*it*_	1.828029		0.029377	0.003690	7.960365		0.0000
x6_*it*_	4.336802		0.068314	0.032365	-2.110754		0.0374
x7_*it*_	2.097449		0.171887	0.103040	1.668156		0.0985
R-squared		1.610179		F-statistic		187.4453	
Adjusted R-squared		0.959210		Prob(F-statistic)		0.000000	
Durbin-Watson stat		1.528659					

*Source: The author compiled the analysis results based on Eviews9.0.*

From the regression results given in Table 8, we can find that the model’s coefficient of determination is 0.964354 and the revised coefficient of determination is 0.959210, indicating that all independent variables have a 95.92% explanatory degree to the dependent variable and the overall fit of the model is good. The F-statistic of the model is 187.4453 and F_*a*_ (k, n-k-1) = F_0.05_ (7,6) = 4.21, when the significance level is a = 0.05, which is less than the F-value. At the same time, the corresponding P-value of the F-test is 0.000000, which passes the F-test at the 1% significance level, indicating that the explanatory variables of the model have high significance on the whole. Finally, the DW value of the model is 1.528659, indicating that the model does not have large spatial autocorrelation problems.

Among them, c_*i*_ (i = 1, 2,…, 8) represents the intercept items corresponding to the eight third-party payment platforms. For example, when i = 1, c_*i*_ = c_1_ = 1.786514, the specific regression equation between Alipay’s pricing to merchants and its influencing factor is as follow:

$$y_{t}=1.7865-0.2355x_{1t}-0.0314x_{2t}+0.0291x_{3t}-0.2476x_{4t}+0.0294x_{5t}+0.0683x_{6t}+0.1719x_{7t}$$

(2) (t=1,2,…,14)

Based on the above-mentioned analysis, the final regression equation is as follows:

$$\begin{array}{l} y_{it}=c_{i}-0.2355x_{1it}-0.0314x_{2it}+0.0291x_{3it}-0.2476x_{4it}+0.0294x_{5it}+0.0683x_{6it}+0.1719x_{7it}\\ \left(-4.328700)(-0.370074)(0.510879\right)\\ \left(-2.021538)(7.960365)(-2.110754)(1.668156\right) \end{array}$$

(3) (i=1,2,…,8;t=1,2,…,14)

The values in parentheses below represent the *t*-test values of the corresponding coefficients. At a given significance level of a = 0.05, the critical value t_*a/2*_ (n-k-1) = t_0.025_ (6) = 1.943. From the regression results of the equation, only the consumer scale x_1*it*_, the merchant’s same-sided network externality x_4*it*_, the platform’s market share x_5*it*_, and the platform’s bank fee cost x_6i_t correspond to |t| <t_0.025_ (6). At the same time, the P-values corresponding to these four variables are 0.0000, 0.0460, 0.0000, and 0.0374, respectively, indicating that these variables have a significant impact on the pricing of third-party payment platforms to merchants. The consumer’s same-sided network externality x_2*it*_, merchant scale x_3*it*_, and the selection of platform business model x_7*it*_ correspond to |t| t_0.025_ (6). At the same time, the P-values corresponding to these three variables are 0.7121, 0.6106, and 0.0985, respectively, indicating that these variables have no significant impact on the pricing of the platform to merchants.

## Conclusion

### Consumer Scale and Platform Pricing to Merchant Research Conclusion

The regression coefficient of the merchant’s network externality is –0.247604, which means that under other conditions unchanged, the strength of the same-sided network externality of the merchant increases by 1%, and the platform pricing for the merchant decreases by 0.247604%. The regression coefficient of consumers is –0.235485, under other conditions unchanged, for every positive change in the number of consumers, the platform pricing to the merchant will change negatively by 0.235485 percentage points. The *T*-test value is –4.328700, |t| > t_0.025_(6) = 1.943, and the corresponding P-value is 0.0000. It shows that there is a negative correlation between the platform’s pricing to merchants and the scale of consumers, and the latter has a significant impact on the former. Therefore, Hypothesis H_1_ is valid. The scale of consumers is the main factor that determines the platform’s pricing to merchants.

### Consumer’s Same-Side Network Externality and Platform Pricing to Merchant

The regression coefficient of consumers’ same-side network externality is –0.031387, which means that when other conditions remain unchanged, for every 1% increase in the strength of consumer’s same-side network externality, the platform’s pricing to merchants will fall by 0.031387%. The *T*-test value is –0.370074, |t|<t_0.025_(6) = 1.943, and the corresponding P-value is 0.7121, and it fails to pass the *T*-test. It shows that there is a negative correlation between the platform pricing to merchants and consumer’s same-side network externality, but the latter has no significant impact on the former. Therefore, Hypothesis H_3_ is valid to a little extent. The impact of the consumer’s same-side network externality on third-party payment platforms and the platform pricing to merchants is not significant. The consumer’s same-side network externality is not the main factor that determines the pricing of the platform to merchants.

### Merchant Scale and Platform Pricing to Merchant

The regression coefficient of the scale of the merchant is 0.029118, which means that under other conditions unchanged, for each positive change in the number of merchant, the platform’s pricing to merchant changes positively by 0.029118 percentage points. The *T*-test value is 0.510879, |t| <t_0.025_(6) = 1.943, and the corresponding P-value is 0.6106, and it fails to pass the *T*-test. It shows that there is a positive correlation between the platform pricing to merchants and the scale of merchants, and the latter has no significant effect on the former. Therefore, Hypothesis H_2_ is invalid. The merchant scale is not the main factor that determines the pricing of the platform to merchants.

### Merchant’s Same-Side Network Externality and Platform Pricing to Merchant

The regression coefficient of the merchant’s same-side network externality is –0.247604, which means that under other conditions unchanged, for every 1% increase in the strength of the merchant’s same-side network externality, the platform’s pricing to merchants will fall by 0.247604%. The *T*-test value is –2.021538, |t| > t_0.025_ (6) = 1.943, and the corresponding P-value is 0.0460, and it passes the *T*-test at the 5% significance level. It shows that there is a negative correlation between the platform’s pricing to merchants and the merchant’s same-sided network externality, and the latter has a significant impact on the former. Therefore, Hypothesis H_4_ is valid. The merchant’s same-sided network externality is the main factor that determines the pricing of the platform to merchants.

### The Market Share of the Platform and Platform Pricing to Merchant

The regression coefficient of the platform transaction is 0.029377, which means if other conditions remain unchanged, the platform’s market share changes by one percentage point in the positive direction, and the platform’s pricing to merchants changes by 0.029377 percentage points in the positive direction. The *T*-test value is 7.960365, |t| > t_0.025_ (6) = 1.943, and the corresponding P-value is 0.0000, and it passes the *T*-test at the 1% significance level. It shows that there is a positive correlation between the platform’s pricing to merchants and the platform’s market share, and the latter has a significant impact on the former. Therefore, Hypothesis H_5_ is valid. The platform’s market share is the main factor that determines the pricing of the platform to merchants.

### Banking Fee Cost of the Platform and Platform Pricing to Merchant

The regression coefficient of the platform’s bank fee cost is -0.068314, indicating that under other conditions unchanged, the platform’s bank fee cost increases by 1%, and the platform’s pricing to merchants decreases by 0.068314. The *T*-test value is –2.110754, |t| > t_0.025_(6) = 1.943, and the corresponding P-value is 0.0374, and it passes the *T*-test at the 5% significance level. It shows that there is a negative correlation between the platform’s pricing to merchants and the platform’s bank fee costs, and the latter has a significant impact on the former. Therefore, Hypothesis H_6_ is invalid. The cost of bank fees is the main factor that determines the pricing of the platform to merchants.

### The Selection of Platform’s Business Model and Platform Pricing for Merchant

The regression coefficient of the selection of platform’s business model is -0.068314. That is to say, when other conditions remain unchanged, platform’s pricing to merchants in the selection of a vertically integrated model is 0.068314% lower than the pricing in the selection of vertically separated model. The *T*-test value is 1.668156, |t| < t_0.025_(6) = 1.943, and the corresponding P-value is 0.0985, and it fails to pass the *T*-test. It shows that there is a negative correlation between the platform’s pricing to merchants and the selection of platform’s business model, but the latter has no significant effect on the former. Therefore, Hypothesis H_7_ is invalid. The selection of platform’s business model is not the main factor that determines the pricing of platform to merchant.

**FIGURE 2:**
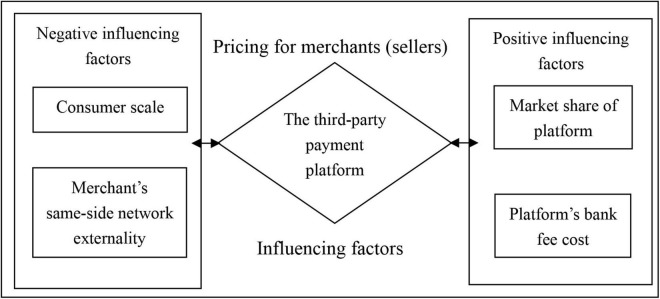
Influencing factors for third-party payment platform pricing to merchant.

The factors that third-party payment platforms should consider when pricing for the merchant are shown in Figure 2.

## Discussion

The theoretical significance of the research in this article mainly includes the following two aspects: On the one hand, we discuss the factors that influence the pricing of third-party payment platform enterprises to merchants connected to the platform and make up for the lack of empirical research on the pricing strategy of third-party payment platform, so as to obtain a relatively complete factor model of pricing of a third-party payment platform to merchants. On the other hand, we analyze the pricing strategies of third-party payment platform enterprises by using the quarterly data of eight payment platforms in China from 2014 to 2017, and observe that in the two-sided market environment, the scale of consumers and the merchant’s same-side network externality have a significant negative impact on the platform’s pricing for merchants. The market share of the platform and the bank fee cost of the platform have positive and significant influence on the platform’s pricing to the merchant. These conclusions make up for the fact that the conclusions simply deduced from economic theory are not consistent with management practice.

The conclusion of this article provides a basis for relevant third-party payment platforms to formulate price strategies and business models. For relevant third-party payment platform enterprises, on the one hand, the key factors that have a significant impact on the pricing of merchants accessing the platform should be clarified, while on the other hand, the interest structure and balance of merchants accessing the platform should be coordinated, so as to seek appropriate ways to maximize the platform profits.

## Research Deficiencies and Prospects

### Research Deficiencies

There are two main limitations in this study. First, this article ignores the impact of other attributes (such as attribution attributes, etc.) of consumers (buyers) and merchants (sellers) on price strategies of third-party payment platform enterprises. The second deficiency is that we need more samples from third-party payment platform enterprises with a larger market. If the research conclusion is extended to other industries, it still needs to be carefully considered.

### Prospects

In future research, we can consider how to build the pricing model of the third-party payment platform enterprises and use the actual operation data or cases of the corresponding enterprises to conduct a more in-depth analysis on the basis of clarifying the factors that influence the pricing of the third-party payment platform. Second, from the conclusions, although the selection of platform business model has no significant impact on the pricing of a third-party payment platform to merchants, the former is also a factor affecting the latter. In the actual operation of the third-party payment platform enterprises, the selection of a business model becomes the main way for the third-party payment platform to participate in market competition. And what will be the impact of different competitive strategies on corporate effectiveness? How platform companies can properly use the business model to participate in market competition to obtain greater market power is also the direction of follow-up research. Finally, the selection of compatible or incompatible competitive strategies is the main problem faced by third-party payment platform companies as they grow to a certain stage. How third-party payment platform companies can properly use exclusive competition methods to participate in market competition, thereby gaining greater market power, also has important theoretical significance and practical value to guide the selection of third-party payment platform companies’ business models.

## Data Availability Statement

The original contributions presented in the study are included in the article/supplementary material, further inquiries can be directed to the corresponding author.

## Author Contributions

NW built the model and conceptualised the writing. WL researched literature and data, and handled the data. JS organized article and wrote the introduction. All authors contributed to the article and approved the submitted version.

## Conflict of Interest

The authors declare that the research was conducted in the absence of any commercial or financial relationships that could be construed as a potential conflict of interest.

## Publisher’s Note

All claims expressed in this article are solely those of the authors and do not necessarily represent those of their affiliated organizations, or those of the publisher, the editors and the reviewers. Any product that may be evaluated in this article, or claim that may be made by its manufacturer, is not guaranteed or endorsed by the publisher.
